# Supplementary material for: Autonomous transposons tune their sequences to ensure somatic suppression
Source: Nature. 2024 Feb 14;626(8001):1116–24. doi: 10.1038/s41586-024-07081-0 (PMC10901741; doi:10.1038/s41586-024-07081-0)
Supplement: Supplementary file 1 — Supplementary Figs. 1–8 and legends for Supplementary Tables 1–15 (tables supplied separately). [file 41586_2024_7081_MOESM1_ESM.pdf]

nature portfolio

https://doi.org/10.1038/s41586-024-07081-0

Supplementary information

Autonomous transposons tune their sequences to ensure somatic suppression

In the format provided by the authors and unedited

Supplementary Figure 1

Figure 2g

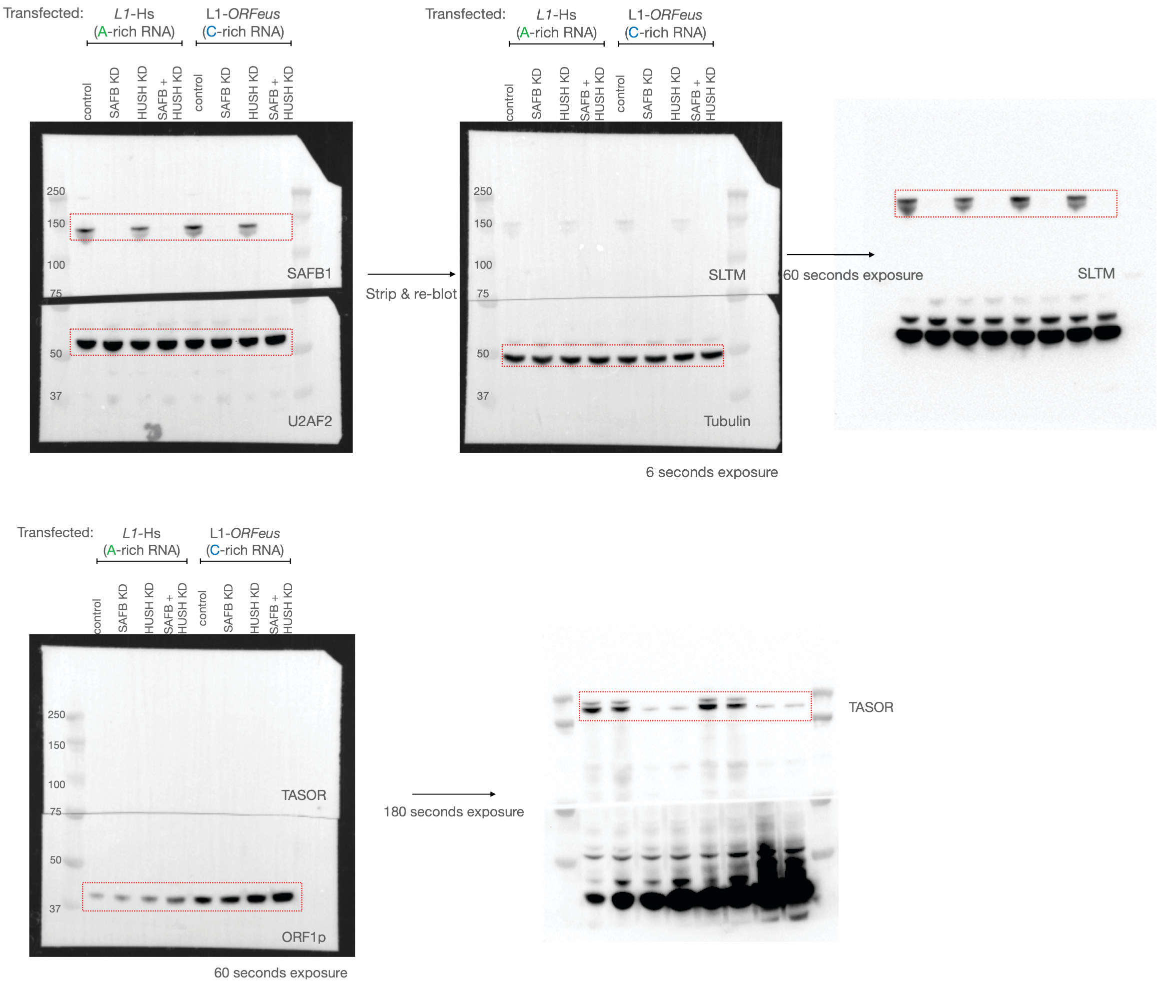

Figure 2h

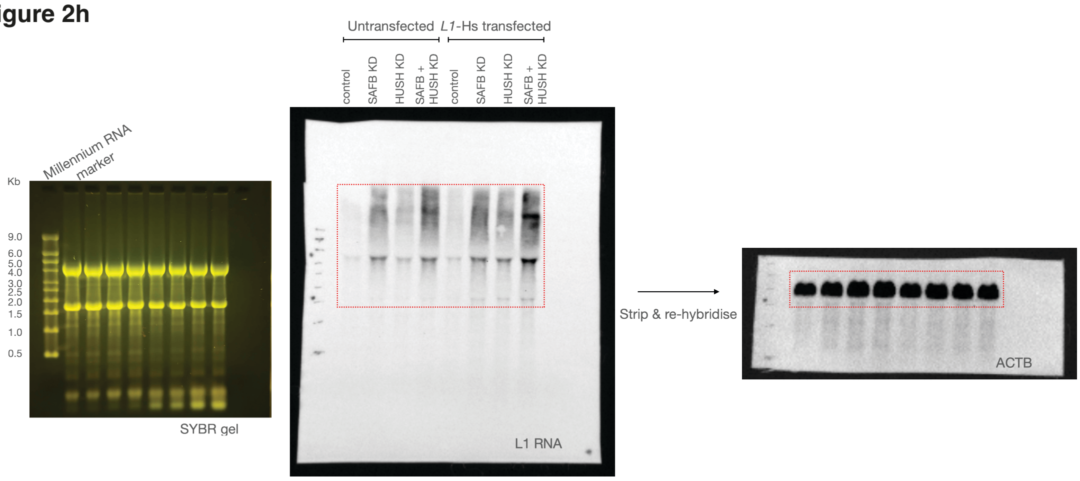

Supplementary Figure 1 (continued)

Extended Data Figure 4b

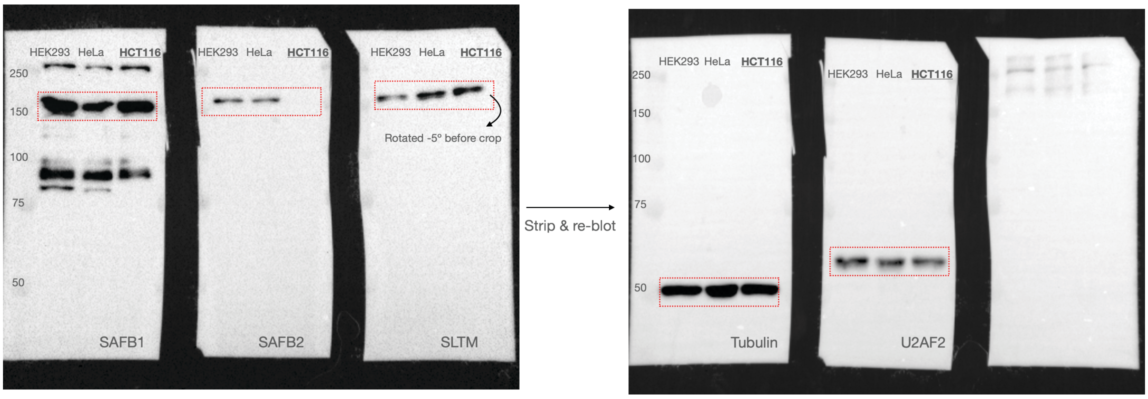

Supplementary Figure 1 (continued)

Extended Data Figure 4c

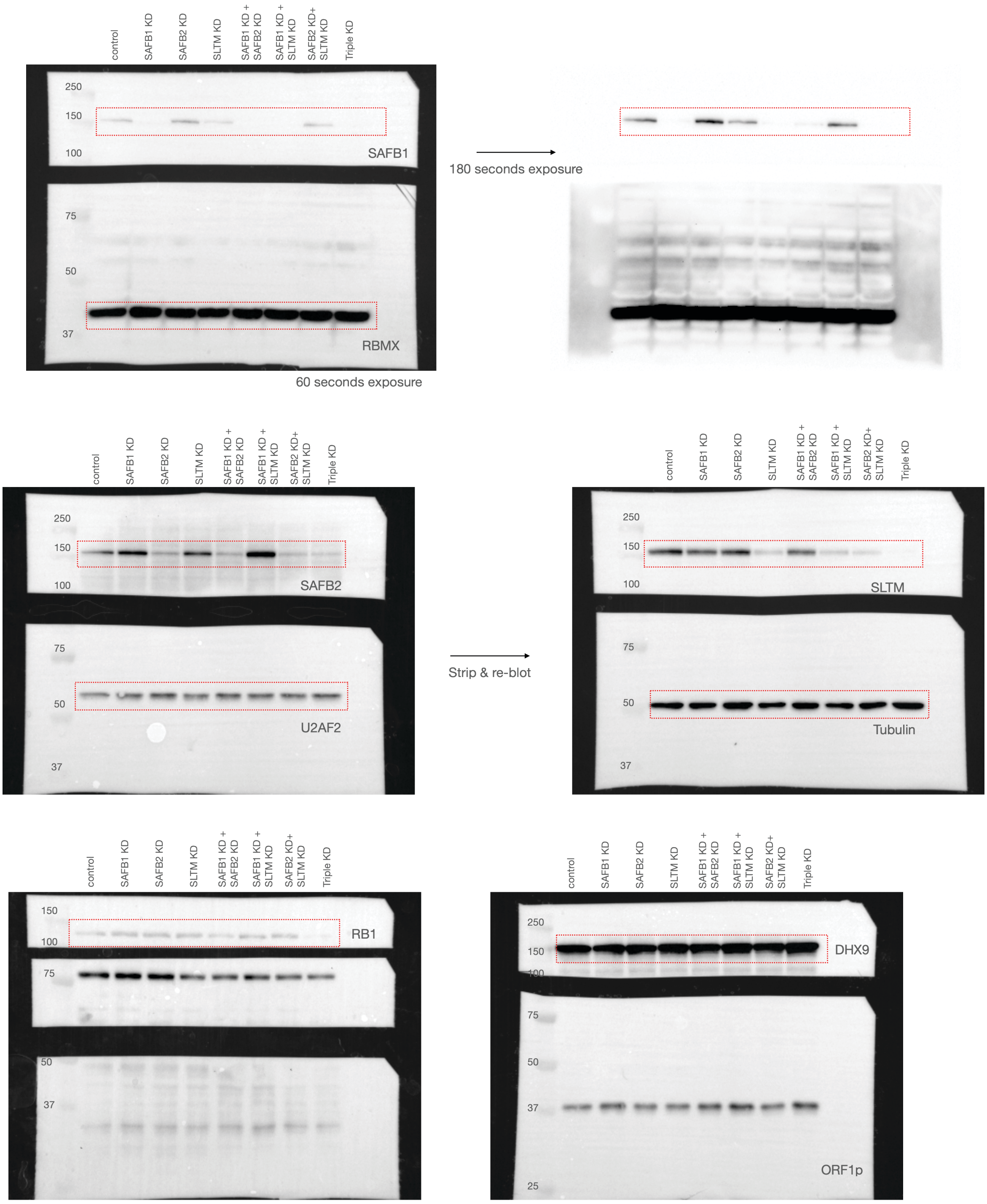

Supplementary Figure 1 (continued)

Extended Data Figure 4d

HCT116 blots:

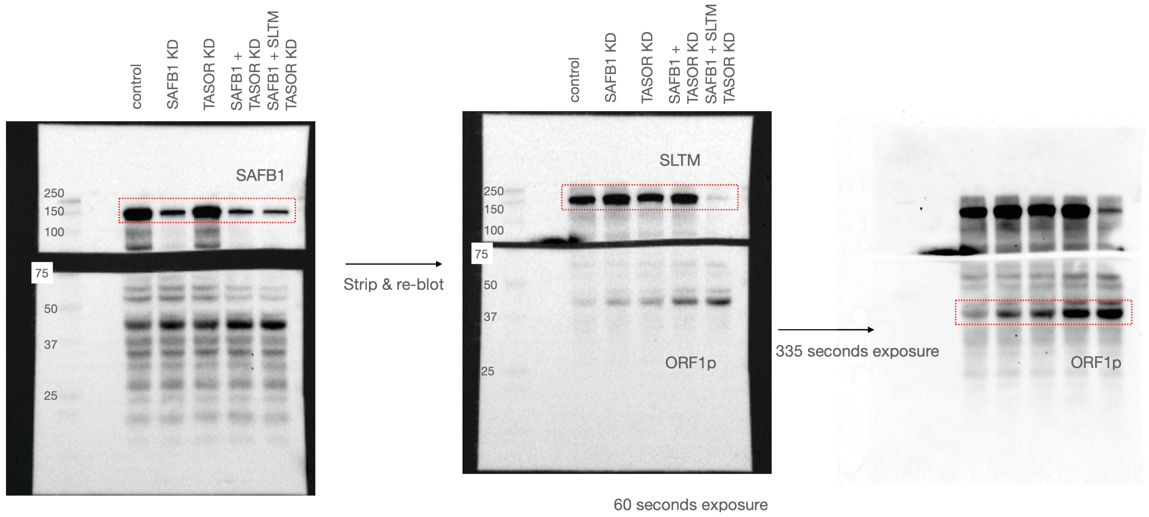

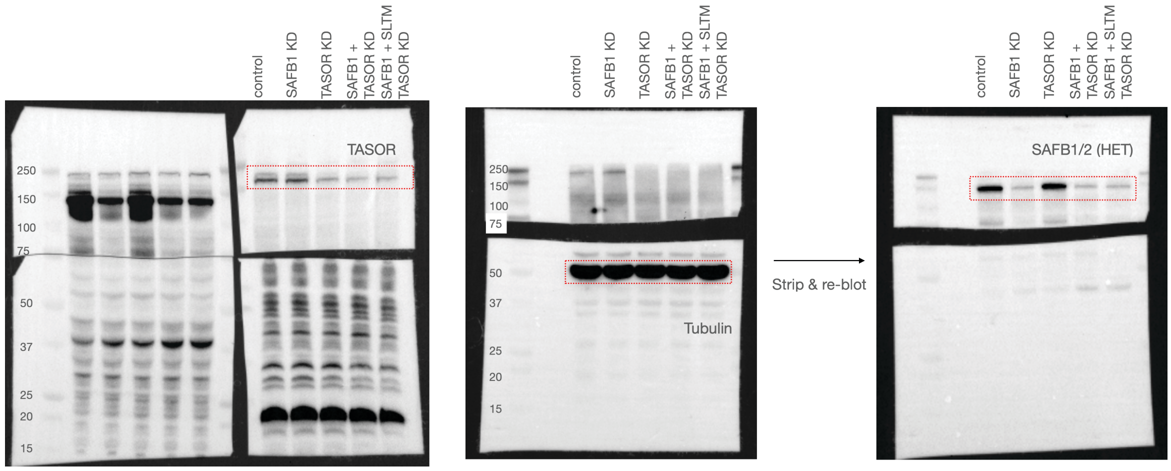

Supplementary Figure 1 (continued)

Extended Data Figure 4d (continued)

HeLa blots:

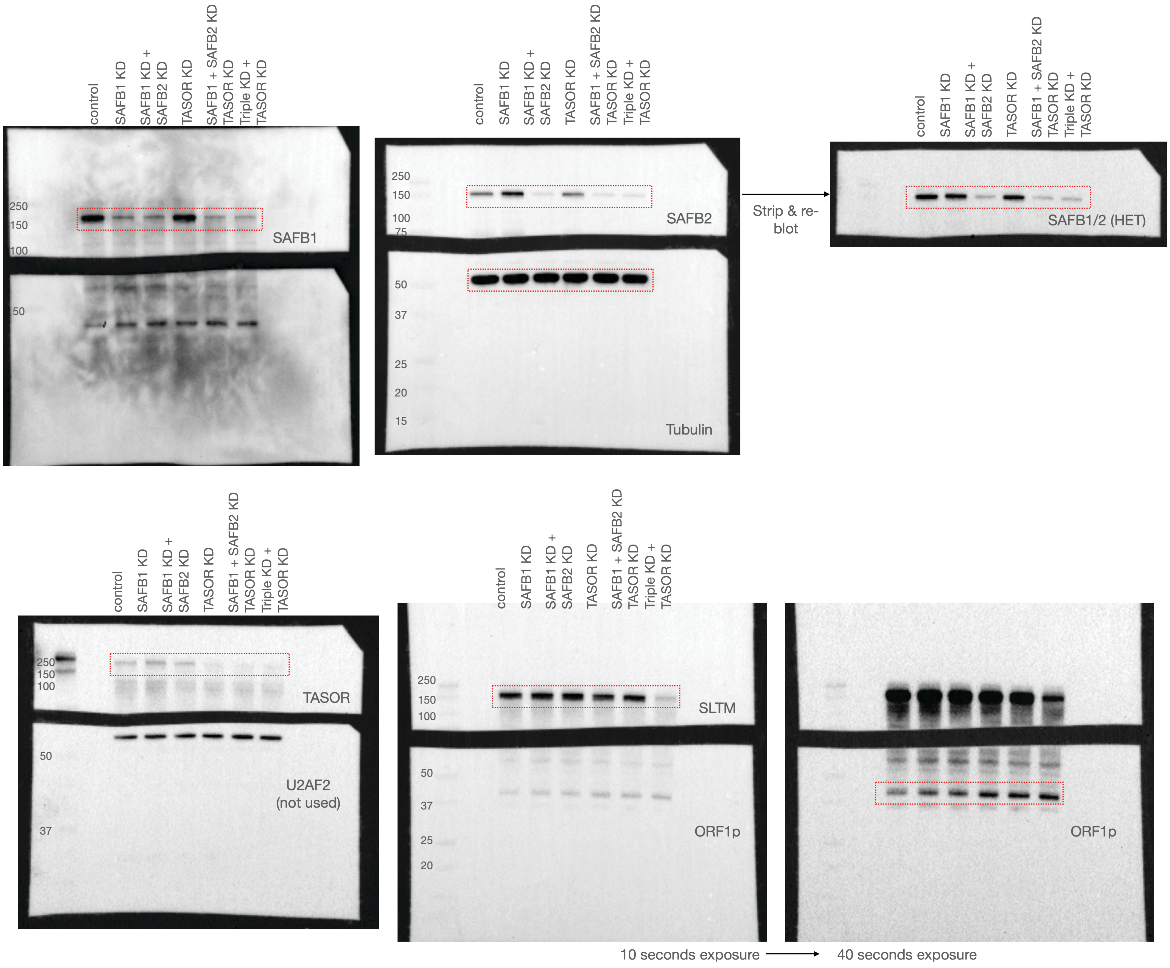

Supplementary Figure 1 (continued)

Extended Data Figure 7b

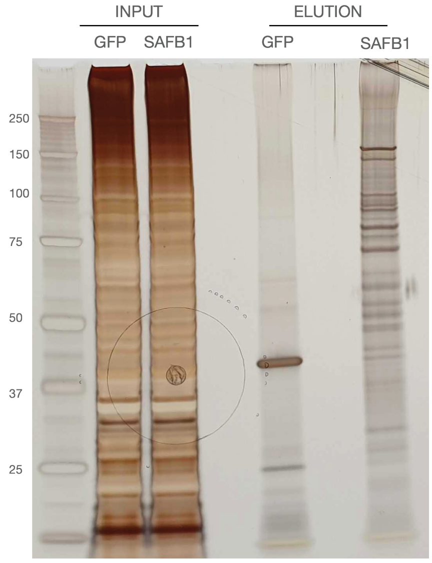

Extended Data Figure 7d

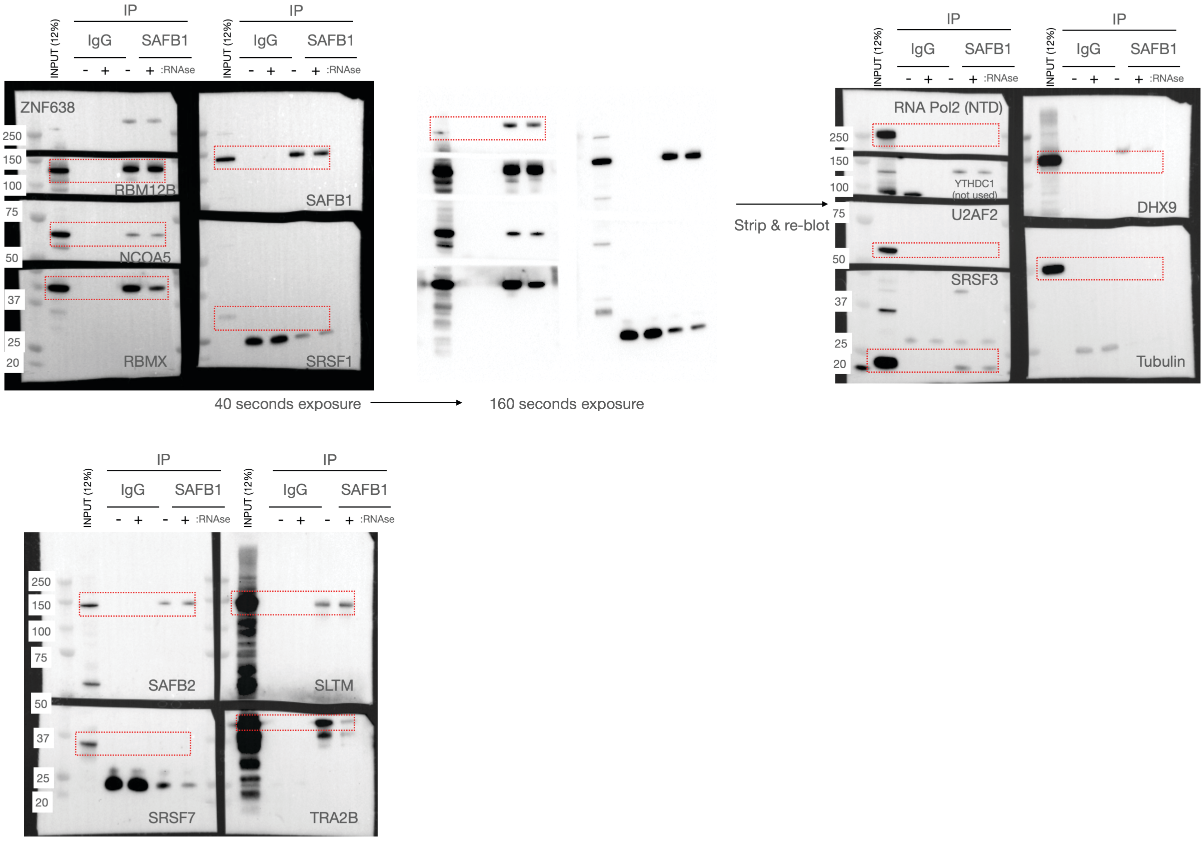

Supplementary Figure 1 (continued)

Extended Data Figure 7c

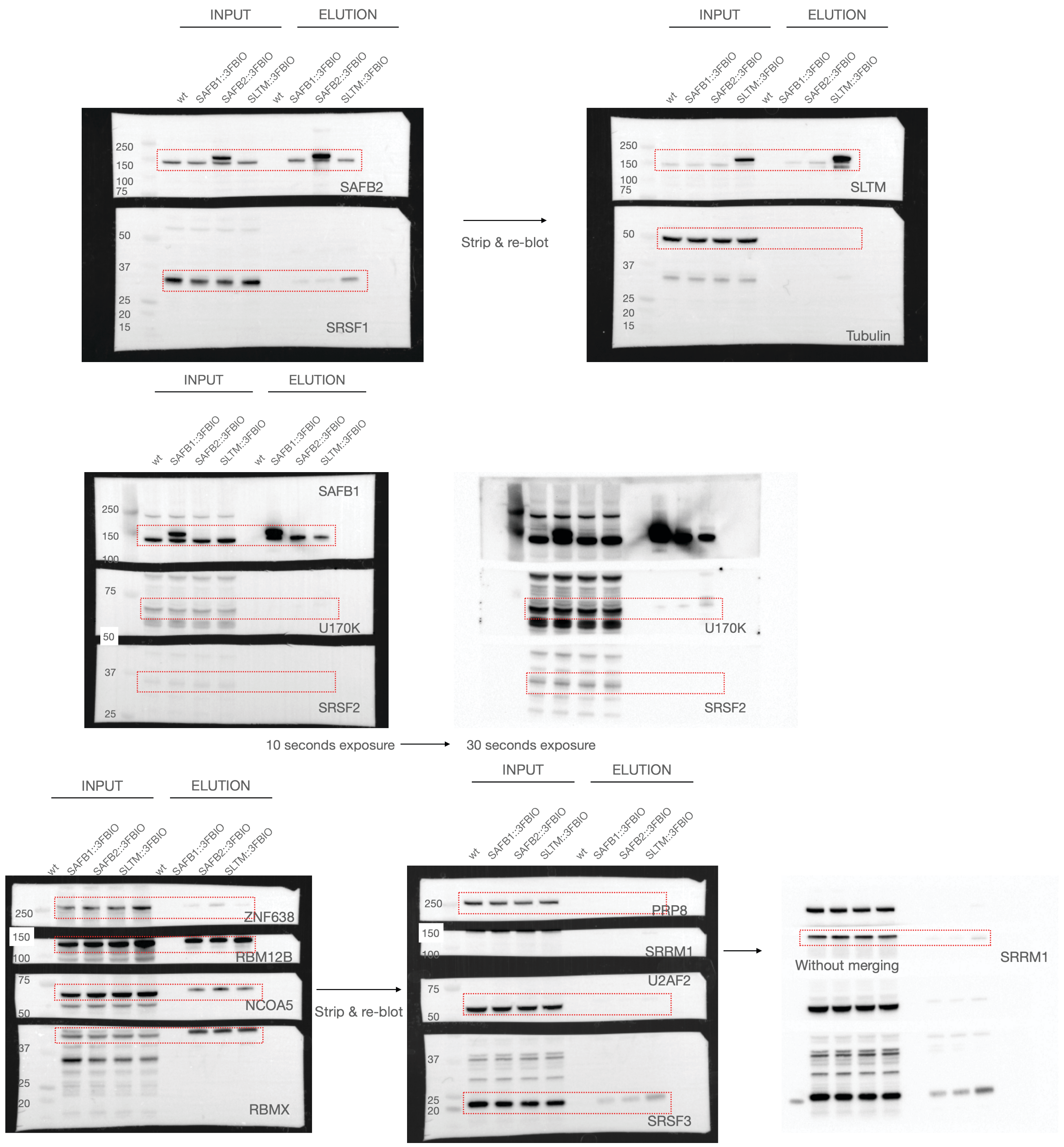

Supplementary Figure 1 (continued)

Extended Data Figure 7c

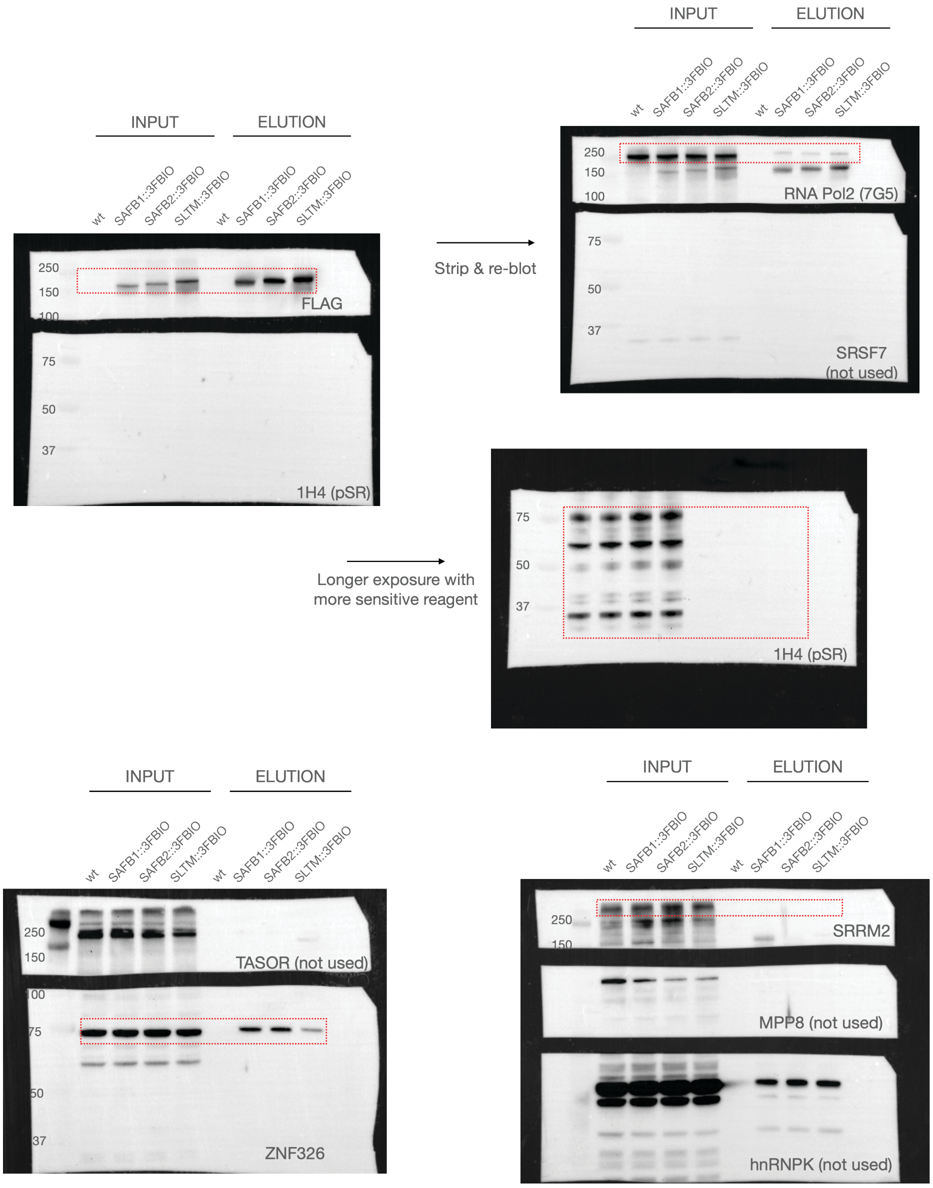

Supplementary Figure 1 (continued)

Extended Data Figure 7e

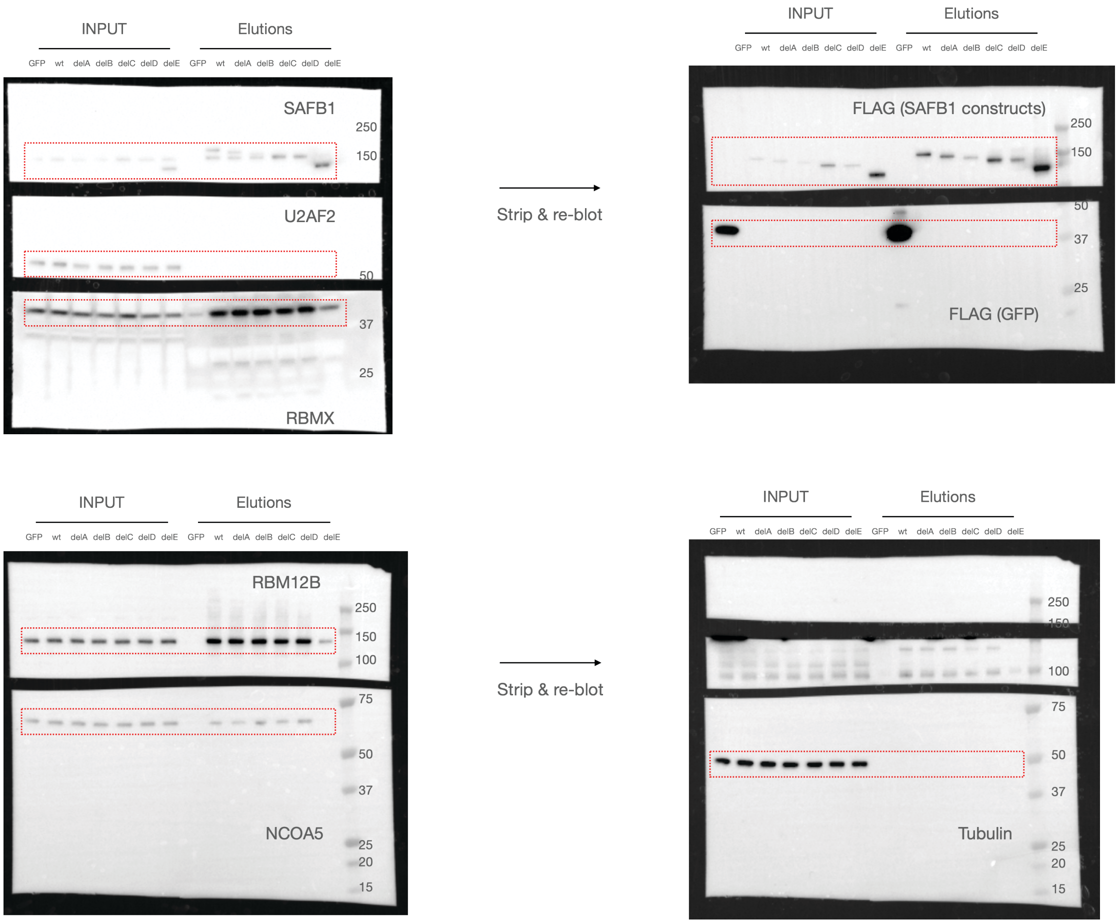

Supplementary Figure 1 (continued)

Extended Data Figure 7g

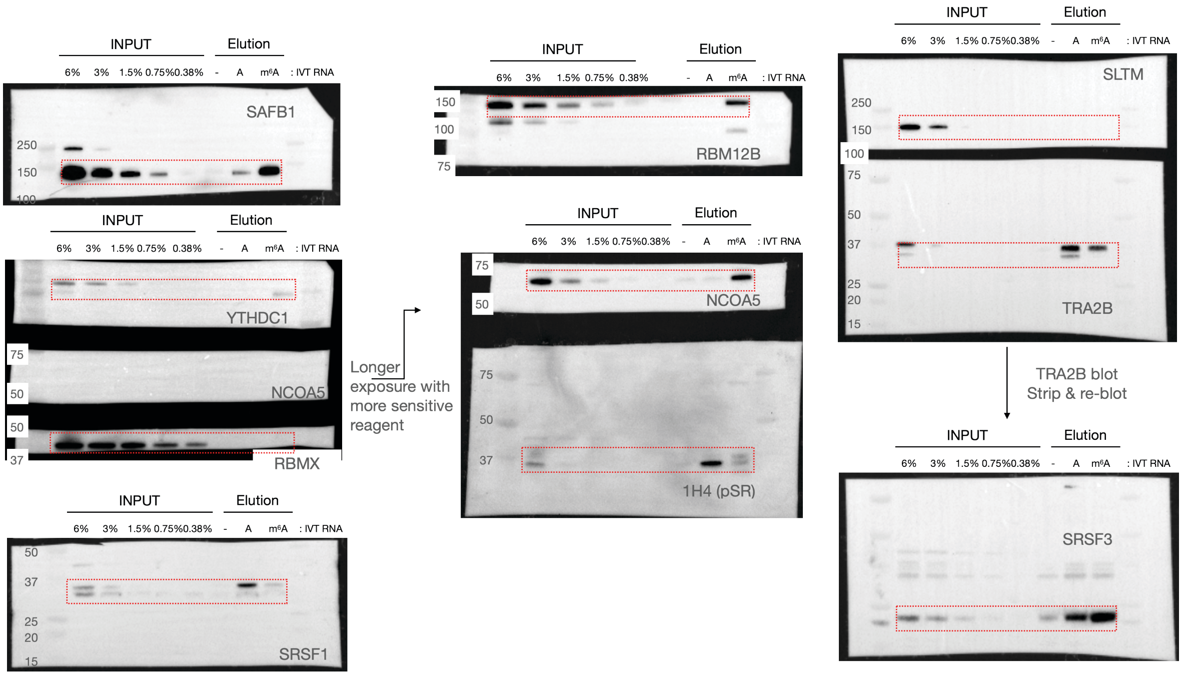

Extended Data Figure 10g

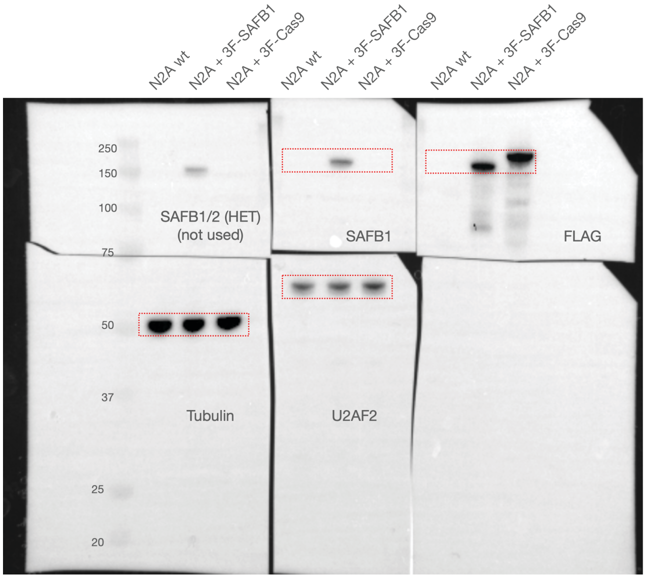

Supplementary Figure 1. Uncropped gels from northern- or western-blots presented in the manuscript.

Supplementary Figure 2

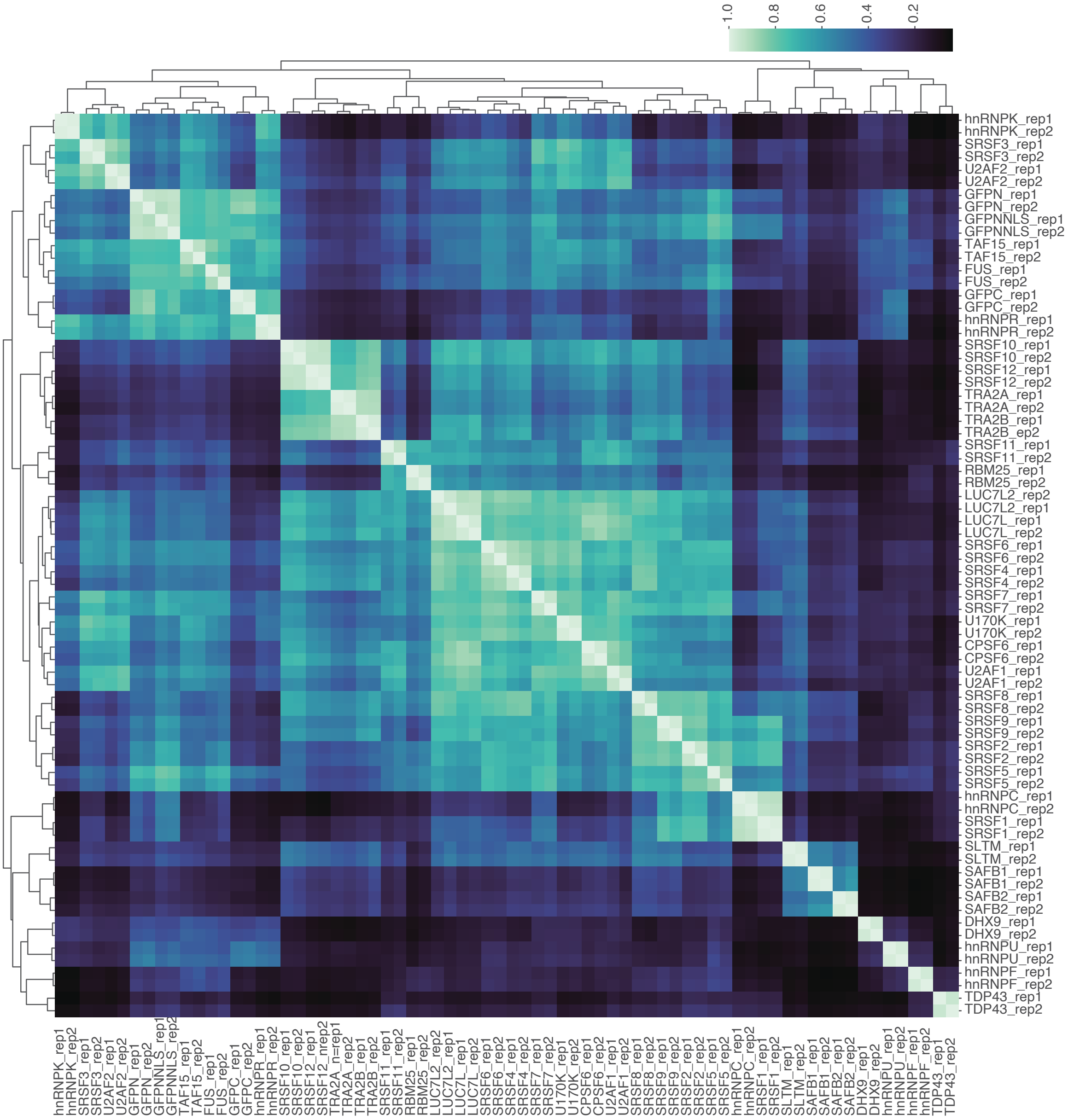

Supplementary Figure 2. Correlation matrix of all RNA-binding proteins profiled using FLASH and negative controls, together with biological replicates.

Supplementary Figure 3

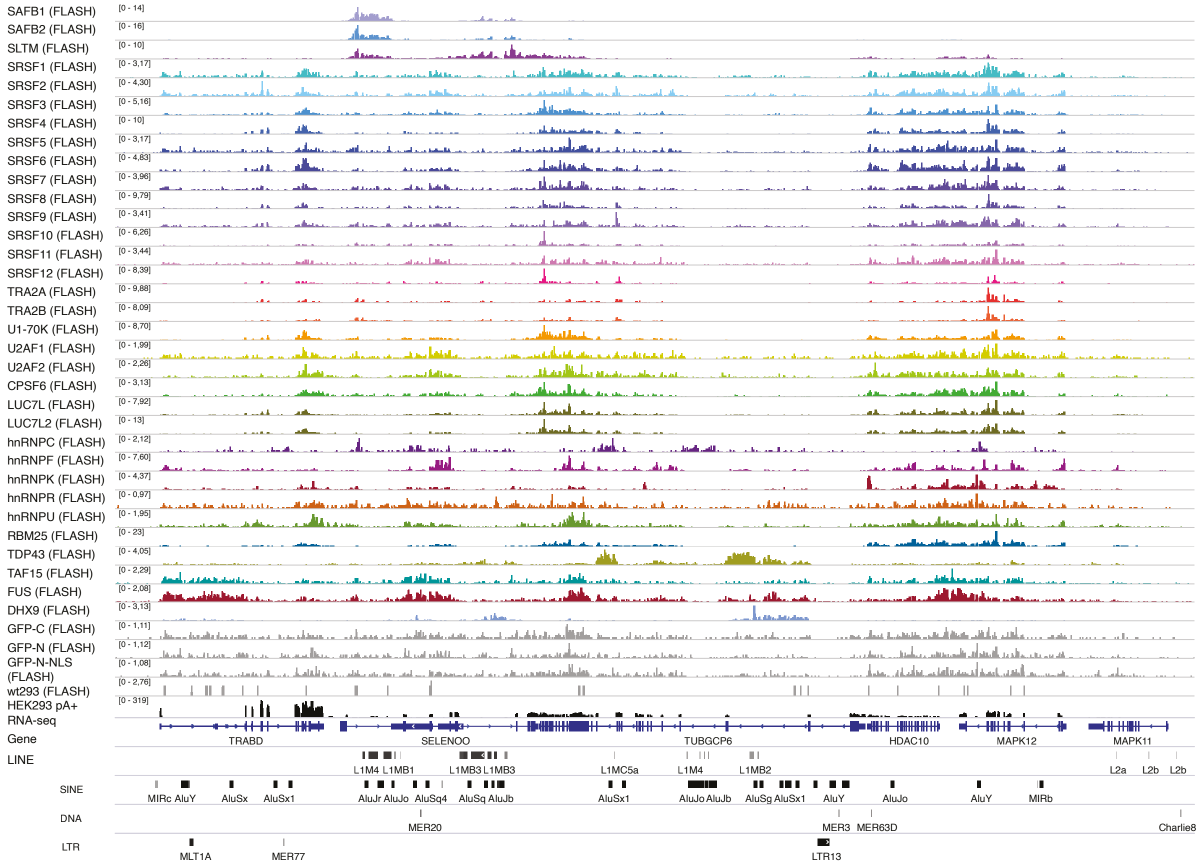

Supplementary Figure 3. Sample locus with coverage of all RBPs profiled using FLASH

Supplementary Figure 4

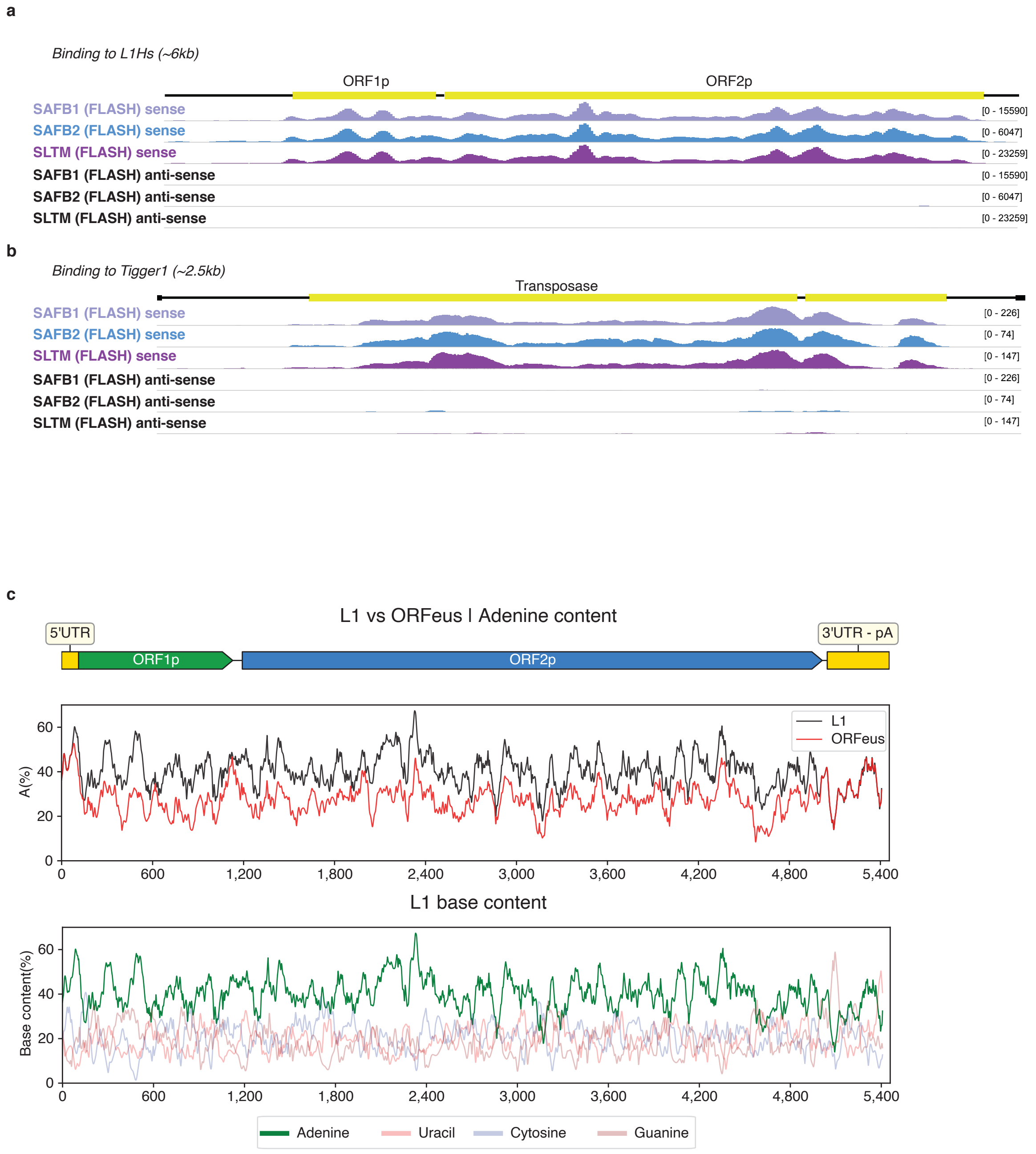

Supplementary Figure 4. SAFB proteins bind to coding regions of autonomous transposons that contain A-bias.
a, FLASH coverage of SAFB1, SAFB2 and SLTM on a model, full-length L1Hs transposon (~6kb) with two protein-coding regions labelled as ORF1p and ORF2p.
b, FLASH coverage of SAFB1, SAFB2 and SLTM on a reconstructed, full-length Tigger1 transposon (~2.5kb) with one protein-coding region labelled as Transposase.
c, Base content of the constructs used in (i). (Top) Comparison of adenine content between wt-L1Hs and ORFeus, which is genetically recoded to remove the A-bias of wt-L1Hs[21]. (Bottom) Relative percentage of all four bases in wt-L1Hs, showing the striking enrichment of adenines (window size = 50nt).

Supplementary Figure 5

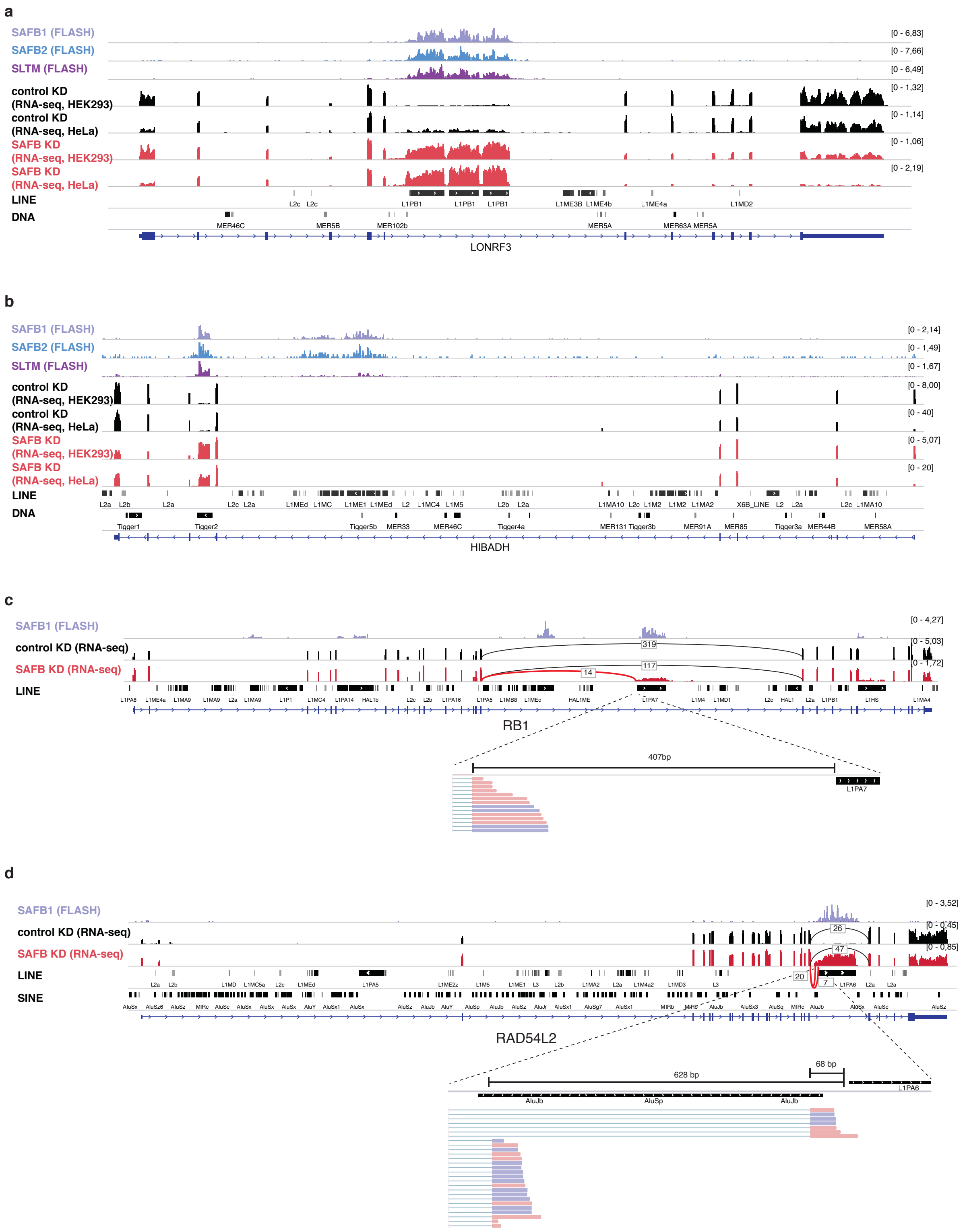

Supplementary Figure 5. SAFB depletion activates cryptic splice sites upstream of bound regions

a, IGV snapshot showing FLASH coverage of SAFB1, SAFB2 and SLTM, as well as RNA-seq coverage in control vs SAFB1 + SAFB2 + SLTM (SAFB) siRNA treated HEK293 and HeLa cells over LONRF3 gene, where an L1PB1 element is bound by SAFB proteins and gets exonized upon SAFB-depletion, truncating gene expression downstream of the insertion.
b, IGV snapshot showing FLASH coverage of SAFB1, SAFB2 and SLTM, as well as RNA-seq coverage in control vs SAFB1 + SAFB2 + SLTM (SAFB) siRNA treated HEK293 and HeLa cells over HIBADH gene where an Tigger2 element is bound by SAFB proteins and gets exonized upon SAFB-depletion, truncating gene expression downstream of the insertion.
c, IGV snapshot showing FLASH coverage of SAFB1, as well as RNA-seq coverage in control vs SAFB1 + SAFB2 + SLTM (SAFB) siRNA treated HEK293 cells over RB1 gene, where an L1PA7 element is bound by SAFB proteins and gets exonized upon SAFB-depletion by activating a splice acceptor site 407nt upstream of the TE.
d, IGV snapshot showing FLASH coverage of SAFB1, as well as RNA-seq coverage in control vs SAFB1 + SAFB2 + SLTM (SAFB) siRNA treated HEK293 cells over RAD54L2 gene, where an L1PA6 element is bound by SAFB proteins and gets exonized upon SAFB-depletion by activating splice acceptor sites 407nt and 68nt upstream of the TE.

Supplementary Figure 6

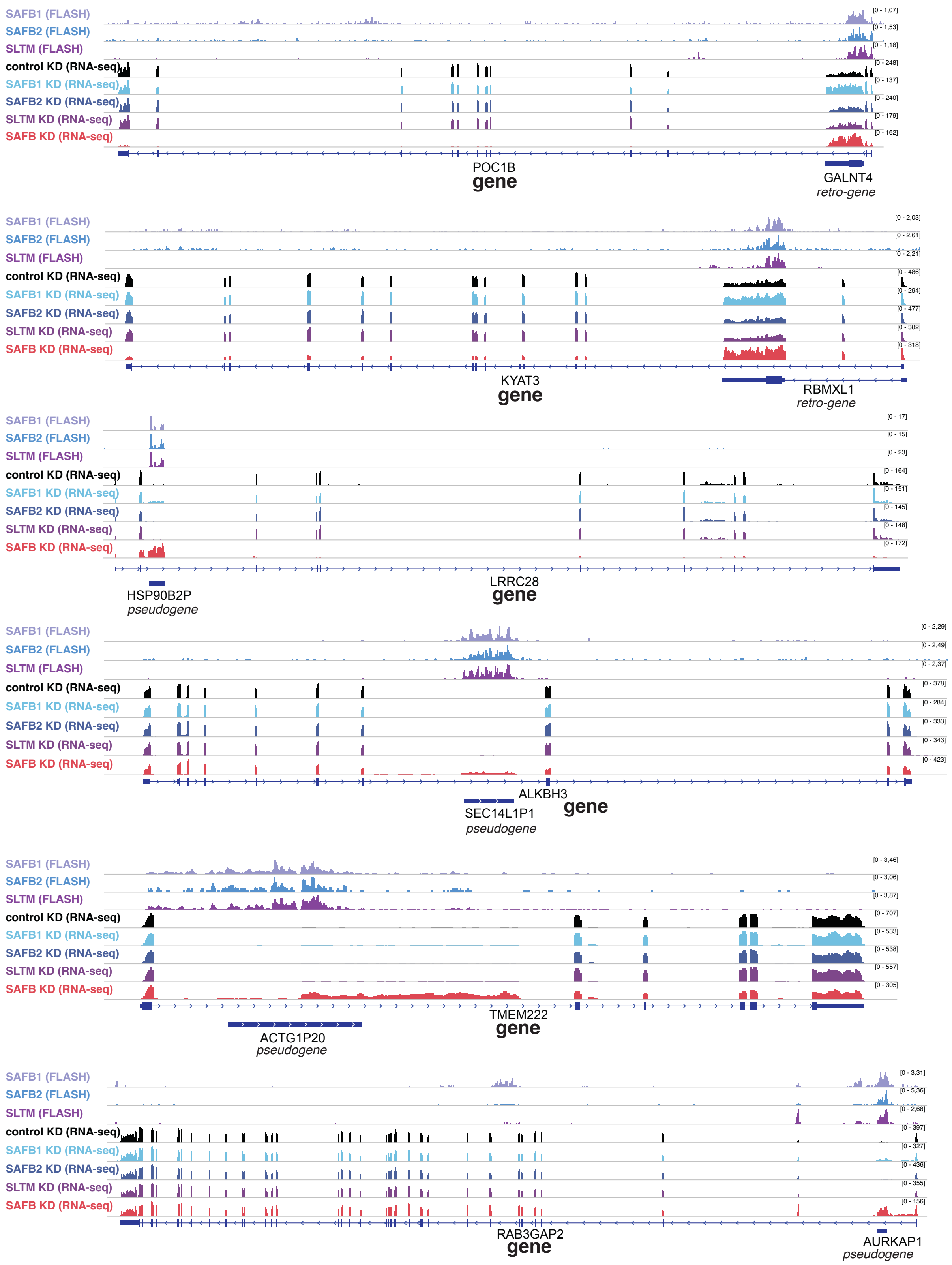

Supplementary Figure 6. SAFB depletion attenuates gene expression by activating splice and/or termination sites within intronic pseudogenes

IGV snapshots showing FLASH coverage of SAFB1, SAFB2 and SLTM, as well as RNA-seq coverage in control, SAFB1, SAFB2, SLTM and SAFB1 + SAFB2 + SLTM (SAFB) siRNA treated HEK293 cells. All pseudogenes are inserted on the same strand as the host gene, and interrupt gene expression, particularly in SAFB-depleted cells. Retro-genes are pseudogenes that are evolutionarily conserved, preserve their coding sequences and thus are more likely to be functional copies of the original genes.

Supplementary Figure 7

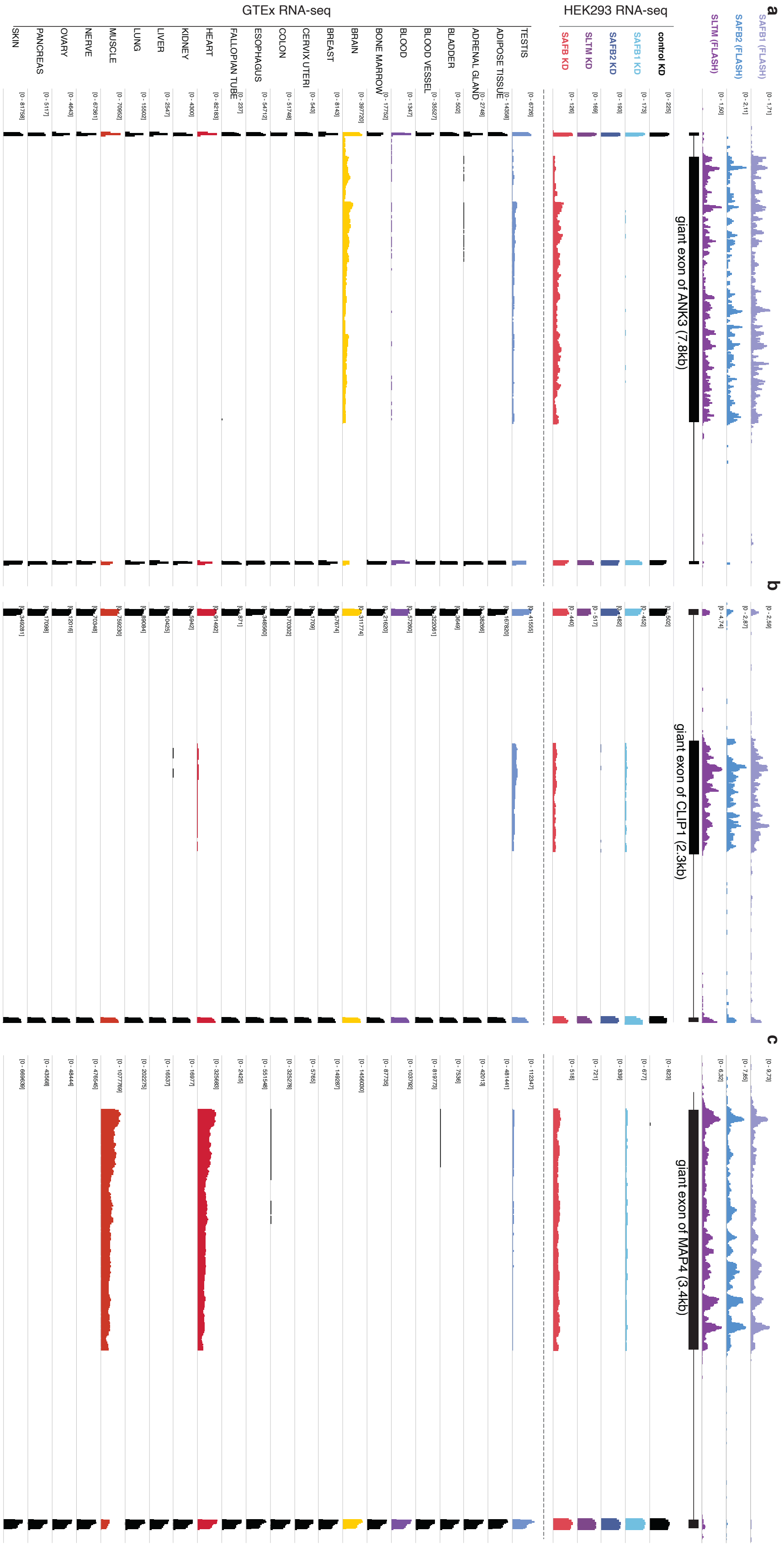

Supplementary Figure 7. SAFB proteins bind to and suppress giant coding exon splicing
IGV snapshot showing FLASH coverage of SAFB1, SAFB2 and SLTM, as well as RNA-seq coverage in control SAFB1, SAFB2, SLTM and SAFB1 + SAFB2 + SLTM (SAFB) siRNA treated HEK293 cells, as well as the cumulative coverage of GTEx data separate by tissues for the giant coding exons of:
a, ANK3
b, CLIP1
c, MAP4.

Supplementary Figure 8

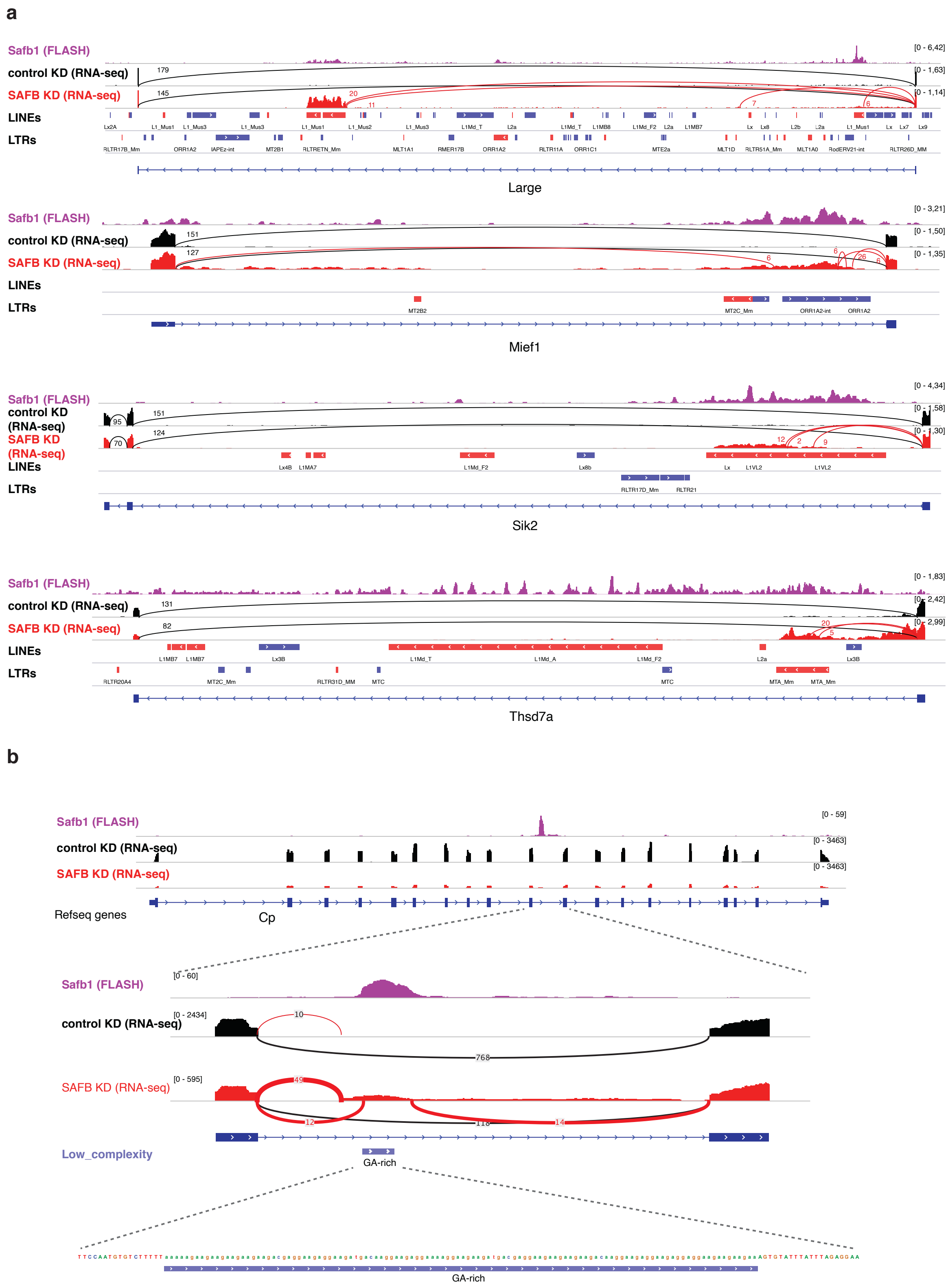

Supplementary Figure 8. L1, LTR and GA-rich simple repeats activate upstream cryptic splice sites in SAFB depleted mouse cells

a, Exonization of L1 and LTR elements in mouse 3T3 cells upon SAFB depletion. Arcs indicate splice junctions between genes and the TE.

b, IGV snapshot showing FLASH coverage of Safb1, as well as RNA-seq coverage in control vs Safb1 + Safb2 + Sltm (SAFB) siRNA treated 3T3 cells over Cp, which is one of the most severely downregulated genes in 3T3 cells upon SAFB-depletion. Zoomed views show that the strong binding of Safb1 coincides with a GA-rich repeat, the preferred motif SAFB proteins interact with (Extended Data Fig. 5l), which leads to activation of both splice acceptor and donor sites in its vicinity. Lines connecting exons designate reads that support that splice junction, novel and/or enhanced splice junctions are shown in red.

Supplementary Table Legends

Supplementary Table 1 (Uploaded as separate excel sheet.)
kmer counts of transposons and SAFB target or non-target giant exons

Supplementary Table 2 (Uploaded as separate excel sheet.)
Oligo sequences used in cloning the constructs, cell line generation, in qPCR experiments, siRNA sequences for human KDs, dsRNA sequences for fly KDs, probe sequences, and IVT RNA used in pulldown experiment.

Supplementary Table 3 (Uploaded as separate excel sheet.)
Results of all the Mass Spectrometry experiments.

Supplementary Table 4 (Uploaded as separate excel sheet.)
TEtranscripts output file for SAFB1 KD in HEK293 polyA RNA-seq.

Supplementary Table 5 (Uploaded as separate excel sheet.)
TEtranscripts output file for SAFB2 KD in HEK293 polyA RNA-seq.

Supplementary Table 6 (Uploaded as separate excel sheet.)
TEtranscripts output file for SLTM KD in HEK293 polyA RNA-seq.

Supplementary Table 7 (Uploaded as separate excel sheet.)
TEtranscripts output file for triple KD in HEK293 polyA RNA-seq.

Supplementary Table 8 (Uploaded as separate excel sheet.)
TEtranscripts output file for triple KD in HeLa polyA RNA-seq.

Supplementary Table 9 (Uploaded as separate excel sheet.)
TEtranscripts output file for triple KD in HCT116 polyA RNA-seq.

Supplementary Table 10 (Uploaded as separate excel sheet.)
TEtranscripts output file for triple KD in HEK293 ribo-depleted Total RNA-seq.

Supplementary Table 11 (Uploaded as separate excel sheet.)
TEtranscripts output file for triple KD in HEK293 nuclear fraction, ribo-depleted Total RNA-seq.

Supplementary Table 12 (Uploaded as separate excel sheet.)
TEtranscripts output file for triple KD in HEK293 cytoplasmic fraction, ribo-depleted Total RNA-seq.

Supplementary Table 13 (Uploaded as separate excel sheet.)
TEtranscripts output file for triple KD in mouse 3T3, polyA RNA-seq.

Supplementary Table 14 (Uploaded as separate excel sheet.)
TEtranscripts output file for Safb KD in Drosophila S2, polyA RNA-seq.

Supplementary Table 15 (Uploaded as separate excel sheet.)
differentially expressed genes with respect to SAFB peaks, log2FC threshold of 1 or 0.
