# Supplementary material for: Autonomous transposons tune their sequences to ensure somatic suppression
Source: Nature. 2024 Feb 14;626(8001):1116–24. doi: 10.1038/s41586-024-07081-0 (PMC10901741; doi:10.1038/s41586-024-07081-0)
Supplement: Supplementary file 2 — Reporting Summary [file 41586_2024_7081_MOESM2_ESM.pdf]

nature portfolio

Corresponding author(s): Tugce Aktas

Last updated by author(s): Jan 3, 2024

Reporting Summary

Nature Portfolio wishes to improve the reproducibility of the work that we publish. This form provides structure for consistency and transparency in reporting. For further information on Nature Portfolio policies, see our Editorial Policies and the Editorial Policy Checklist.

Statistics

For all statistical analyses, confirm that the following items are present in the figure legend, table legend, main text, or Methods section.

n/a	Confirmed	
☐	☒	The exact sample size (*n*) for each experimental group/condition, given as a discrete number and unit of measurement
☐	☒	A statement on whether measurements were taken from distinct samples or whether the same sample was measured repeatedly
☐	☒	The statistical test(s) used AND whether they are one- or two-sided<br>*Only common tests should be described solely by name; describe more complex techniques in the Methods section.*
☒	☐	A description of all covariates tested
☐	☒	A description of any assumptions or corrections, such as tests of normality and adjustment for multiple comparisons
☐	☒	A full description of the statistical parameters including central tendency (e.g. means) or other basic estimates (e.g. regression coefficient) AND variation (e.g. standard deviation) or associated estimates of uncertainty (e.g. confidence intervals)
☐	☒	For null hypothesis testing, the test statistic (e.g. *F*, *t*, *r*) with confidence intervals, effect sizes, degrees of freedom and *P* value noted<br>*Give P values as exact values whenever suitable.*
☒	☐	For Bayesian analysis, information on the choice of priors and Markov chain Monte Carlo settings
☒	☐	For hierarchical and complex designs, identification of the appropriate level for tests and full reporting of outcomes
☒	☐	Estimates of effect sizes (e.g. Cohen's *d*, Pearson's *r*), indicating how they were calculated

Our web collection on statistics for biologists contains articles on many of the points above.

Software and code

Policy information about availability of computer code

Data collection	Deep sequencing was performed with NovaSeq (Illumina). Long read direct RNA sequencing was performed with ONT. Luciferase activity was read on Omega Lumistar machine. Confocal microscopy images are acquired with Leica Stellaris 8.
Data analysis	High-throughput sequencing data were analyzed with: bowtie2 (v. 2.3.5), cutadapt 4.1, STAR 2.7.9a aligner, UCSC repeatMasker annotation, HOMER, rMATs, DESeq2, DEXSeq , Splice AI, recount3. The data analysis is described in the "Methods" section and code is available at: https://github.com/aktas-lab/safb_paper

For manuscripts utilizing custom algorithms or software that are central to the research but not yet described in published literature, software must be made available to editors and reviewers. We strongly encourage code deposition in a community repository (e.g. GitHub). See the Nature Portfolio guidelines for submitting code & software for further information.

Data

Policy information about availability of data

All manuscripts must include a data availability statement. This statement should provide the following information, where applicable:

- Accession codes, unique identifiers, or web links for publicly available datasets
- A description of any restrictions on data availability
- For clinical datasets or third party data, please ensure that the statement adheres to our policy

The FLASH and RNA-seq data were deposited in the Gene Expression Omnibus under accession code: GSE223263

Human research participants

Policy information about studies involving human research participants and Sex and Gender in Research.

Reporting on sex and gender	n/a
Population characteristics	n/a
Recruitment	n/a
Ethics oversight	n/a

Note that full information on the approval of the study protocol must also be provided in the manuscript.

Field-specific reporting

Please select the one below that is the best fit for your research. If you are not sure, read the appropriate sections before making your selection.

☒ Life sciences ☐ Behavioural & social sciences ☐ Ecological, evolutionary & environmental sciences

For a reference copy of the document with all sections, see nature.com/documents/nr-reporting-summary-flat.pdf

Life sciences study design

All studies must disclose on these points even when the disclosure is negative.

Sample size	No statistical methods were used to predetermine sample size.
Data exclusions	No data were excluded form the analysis.
Replication	Experiments were repeated as described in the methods section. All experiments shown could be reproduced as described.
Randomization	This study does not involve animal experiments (only tissues sections were taken from the male wild-type or Dnmt3C KO animals)
Blinding	The experiments presented in this article were not blinded (consistent with what is published in the field).

Reporting for specific materials, systems and methods

We require information from authors about some types of materials, experimental systems and methods used in many studies. Here, indicate whether each material, system or method listed is relevant to your study. If you are not sure if a list item applies to your research, read the appropriate section before selecting a response.

Materials & experimental systems

n/a	Involved in the study	
☐	☒	Antibodies
☐	☒	Eukaryotic cell lines
☒	☐	Palaeontology and archaeology
☐	☒	Animals and other organisms
☒	☐	Clinical data
☒	☐	Dual use research of concern

Methods

n/a	Involved in the study	
☒	☐	ChIP-seq
☒	☐	Flow cytometry
☒	☐	MRI-based neuroimaging

Antibodies

Antibodies used

In imaging: SAFB1/2 het (Human; Millipore, 05-588 (clone 6F7), Mouse; LSBio LS-C2886411), SLTM (Invitrogen PA5-59154), SON (Sigma, HPA023535), Orf1p (Mouse; Abcam, ab216324)
In Western Blots: SAFB1 (Santa Cruz, sc-393403), SAFB2 (Santa Cruz, sc-514963), SAFB1/2 (HET) (Human; Merck/Sigma-Aldrich, sc05-588), SLTM (Invitrogen, PA5-59154), ORF1p (Human; Abcam, ab230966), TASOR (Sigma-Aldrich, HPA006735), 1H4 (p-SR) (Merck/Sigma-Aldrich, MABE50), RBM12B (Bethyl, A305-871A-T), RBMX (Cell Signalling Technology, 14794S), NCOA5 (Bethyl, A300-790A-T), ZNF638 (Sigma-Aldrich, ZRB1186), ZNF326 (Santa Cruz, sc-390606), TRA2B (Bethyl, A305-011A-M), U2AF2 (U2AF65) (Santa Cruz, sc-53942), TUBULIN (Santa Cruz, sc-32293), SRRM1 (Abcam, ab221061), SRRM2 (SC35) (Sigma-Aldrich, S4045), SON (Sigma-Aldrich, HPA023535), DHX9 (Abcam, ab 183731), U1-70K (SySy, 203011), PRP8 (Santa Cruz, sc-55533), RNAPII (Creative

	Biolabs, CBMAB-XB0938-YC), IgG normal mouse (Santa Cruz, sc-2025), SRSF1 (Santa Cruz, sc-33652), SRSF2 (Abcam, ab204916), SRSF3 (Elabscience, E-AB-32966), SRSF7 (MBL, RN079PW), RB1 (Cell Signalling Technology, 9309S), TRA2B (Santa Cruz, sc-166829) YTHDC1 (Proteintech, 14392-1-AP).
Validation	anti-FLAG-M2 (Sigma) was used for the validation of mouse and fly cell lines that contain endogenously tagged SafB allele. Specificity for SAFB antibodies were validated by siRNA based knock-downs.

Eukaryotic cell lines

Policy information about cell lines and Sex and Gender in Research

Cell line source(s)	Cell lines (human FlpIn Trex HEK293, human HeLa, human HCT116, mouse FlpIn 3T3, mouse N2A, fly S2R+) were all purchased from vendors, repositories or provided by colleagues (as described in the methods section).
Authentication	No further authentication of the cell lines was done after purchasing.
Mycoplasma contamination	Routine mycoplasma contamination tests were performed with Jena Biosciences Mycoplasma (PCR-based) detection kit (PP-401)
Commonly misidentified lines (See ICLAC register)	The list of cell lines used in this study are not amongst the misidentified cell lines.

Animals and other research organisms

Policy information about studies involving animals; ARRIVE guidelines recommended for reporting animal research, and Sex and Gender in Research

Laboratory animals	Dnmt3C knockout animals were generated as described in (Wang et al., 2013).The founder mutation was subsequently backcrossed into the C57BL/6J background. Homozygous knockout males were validated as infertile, with significantly smaller and disordered testes by P42 as reported previously (Barau et al., 2016).
Wild animals	n/a
Reporting on sex	The experiments needed to be performed in a tissue where the SAFB expression levels are dynamically changing, the testis tissue was selected. Therefore, only male mice at p25 or p50 stages were used in this study.
Field-collected samples	n/a
Ethics oversight	The generation of these experimental animals is regulated following ethical review by the Yale University Institutional Animal Care and Use Committee (IACUC, Protocol #2020-20357) and was performed according to governmental and PHS requirements.

Note that full information on the approval of the study protocol must also be provided in the manuscript.
